# Switching between Successful and Dead-End Intermediates in Membrane Fusion

**DOI:** 10.3390/ijms18122598

**Published:** 2017-12-02

**Authors:** Rodion J. Molotkovsky, Timur R. Galimzyanov, Irene Jiménez-Munguía, Konstantin V. Pavlov, Oleg V. Batishchev, Sergey A. Akimov

**Affiliations:** 1 Laboratory of Bioelectrochemistry, A.N. Frumkin Institute of Physical Chemistry and Electrochemistry, Russian Academy of Sciences, 31/4 Leninskiy Prospekt, 119071 Moscow, Russia; swinka87@gmail.com (R.J.M.); timur.galimzyanov@gmail.com (T.R.G.); olegbati@mail.ru (O.V.B.); 2 Department of Theoretical Physics and Quantum Technologies, National University of Science and Technology “MISiS”, 4 Leninskiy Prospekt, 119049 Moscow, Russia; 3 Department of Engineering of Technological Equipment, National University of Science and Technology “MISiS”, 4 Leninskiy Prospekt, 119049 Moscow, Russia; w0r3ss@gmail.com; 4 Laboratory of Electrophysiology, Federal Clinical Center of Physical-Chemical Medicine of FMBA, 1a Malaya Pirogovskaya Street, 119435 Moscow, Russia; qpavlov@mail.ru; 5 Department of Physics of Living Systems, Moscow Institute of Physics and Technology (State University), 9 Institutskiy Lane, 141700 Dolgoprudniy Moscow Region, Russia

**Keywords:** membrane fusion, viral fusion, enveloped viruses, fusion peptides, stalk mechanism, dead-end state

## Abstract

Fusion of cellular membranes during normal biological processes, including proliferation, or synaptic transmission, is mediated and controlled by sophisticated protein machinery ensuring the preservation of the vital barrier function of the membrane throughout the process. Fusion of virus particles with host cell membranes is more sparingly arranged and often mediated by a single fusion protein, and the virus can afford to be less discriminative towards the possible different outcomes of fusion attempts. Formation of leaky intermediates was recently observed in some fusion processes, and an alternative trajectory of the process involving formation of π-shaped structures was suggested. In this study, we apply the methods of elasticity theory and Lagrangian formalism augmented by phenomenological and molecular geometry constraints and boundary conditions to investigate the traits of this trajectory and the drivers behind the choice of one of the possible scenarios depending on the properties of the system. The alternative pathway proved to be a dead end, and, depending on the parameters of the participating membranes and fusion proteins, the system can either reversibly enter the corresponding “leaky” configuration or be trapped in it. A parametric study in the biologically relevant range of variables emphasized the fusion protein properties crucial for the choice of the fusion scenario.

## 1. Introduction

**Figure 1:**
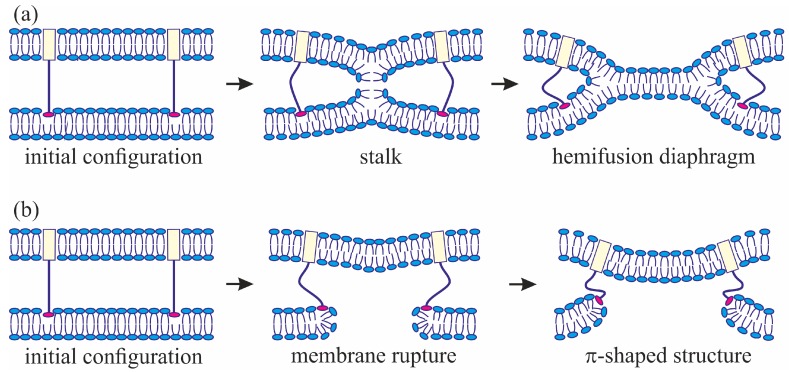
Schematic representation of two scenarios of influenza virus fusion with the host cell membrane: (**a**) a trajectory involving the interim stage of stalk formation, leading to fusion of contact monolayers and formation of a hemifusion diaphragm; (**b**) a trajectory leading to reorientation of the fusion peptides at the edge of the pore and formation of a leaky structure (π-shaped structure). The viral membrane is on the top, the host cell membrane—at the bottom. Transmembrane domains are represented by light-yellow rectangles, fusion peptides—by red ellipses.

Membrane fusion plays an important role in multiple biological processes, such as exocytosis, fertilization, synaptic transmission etc. [1]. Besides these processes normal for a living cell, it also constitutes a crucial stage in viral infection [2]. A commonly adopted paradigm is that any physiological processes involving the remodeling of a biological membrane must not violate its primary function as a physical barrier separating the cell and its organelles from the exterior. Therefore, the stalk-fusion diaphragm model was proposed for description of the rearrangement of the interacting lipid bilayers in the course of fusion [3]. The model assumes that, at the initial stage of fusion, the contact monolayers of the membranes locally merge to form an hourglass-shaped structure (stalk) (Figure 1a), which then expands to bring the distal monolayers into contact and form the so-called trilamellar structure, or hemifusion diaphragm [4] (Figure 1a). Formation of a pore in the hemifusion diaphragm constitutes the final state of fusion. According to theoretical estimates made in a number of publications [5,6,7], the energy barrier to be crossed for stalk formation is estimated at about 40 *k_B_T* (*k_B_T* ~ 4 × 10^−21^ J). This means that, without an additional impact on the system, the membrane would not merge within a physically reasonable time ranging from a few seconds to a few tens of seconds. In biological systems, special proteins known as fusion proteins are responsible for this additional intervention into the system [2,8]. The most thoroughly characterized fusion proteins are those of the synaptic system, participating in the process of Ca^2+^-dependent exocytosis [9,10], and fusion proteins of various envelope viruses, such as influenza virus [11,12,13], HIV [14,15], vesicular stomatitis virus [16], etc.

By contrast with the fusion process associated with synaptic transmission, where the fusion process is mediated by a relatively large ensemble of different proteins, viral fusion is often mediated by a single protein [17], e.g., hemagglutinin (HA) in the case of influenza A virus. The protein forms trimers and consists of a transmembrane (TM) domain and an ectodomain protruding out of the lipid membrane of the virus. Besides this, a fusion peptide is accommodated in a hydrophobic pocket of the ectodomain. The peptide incorporates into the target membrane during a pH-dependent conformational transition of hemagglutinin occurring in the process of viral infection in the cellular endosomes. Subsequent rearrangement of the HA conformation results in close apposition of the viral membrane and the target membrane, bringing the TM domains and fusion peptides into close proximity [18,19]. Coordinated action of several HA proteins is known to be needed for successful fusion in order to form the so-called fusion rosette [20,21,22]. Three major classes of fusion proteins are usually discriminated [23,24] based on their ectodomain structure, and mostly the structure and mechanism of insertion of the fusion peptides into the host cell membrane [25]. However, whether the fusion subunit has a prominent central α-helical coiled-coil (Class I viral fusion proteins), whether it primarily or exclusively consists β-structure (Class II viral fusion proteins), or whether it displays a combination of α-helical and β-structures (Class III viral fusion proteins), the fusion subunits ultimately fold back into a trimer-of-hairpins, in which three C-terminal regions pack on the outside of a central N-terminal trimeric core. When the fold-back is complete, the fusion peptide and the transmembrane anchor are brought together, thereby pulling the two attached membranes (target cell and viral, respectively) together and facilitating their merger [23]. The information on the phenomenology of the viral fusion we used in this work is related to influenza and HIV fusion proteins belonging to class I, since this class is especially well investigated. However, the fusion peptide model we use in our analysis is that of a generalized inclusion into the host membrane, irrespective of the secondary structure details. The only implied assumption is the mechanism whereby the viral and host cell membranes are forced into close proximity by the fusion protein complexes, which is common for all the three classes. As a result, in the approximation of our model, the three classes are essentially equivalent; the only variable being the depth of insertion of the fusion peptide into the host cell membrane and the lateral dimensions of the inserted fragment.

The change of the membrane topology associated with fusion is believed to be impossible achieve by merely breaking the bilayers and then reclosing the system with a topology. The fusion is rather assumed to occur without contact of the cytoplasm with the external environment. Any long-term deterioration of barrier function is a manifestation of a pathological process [26]. However, experimental evidence suggests that a pore piercing the membrane can sometimes be formed at the early stages of viral fusion in one of the membranes [27]. Formation of the through pores at the early stages of membrane fusion was also observed during synaptic vesicle exocytosis or yeast mating [28]. Molecular dynamic simulations also corroborate the possibility of fusion accompanied by leak at the early stage [29,30], as well as the calculations based on the self-consistent field model [31].

The geometry of the possible leaky structure had been unknown until very recently. However, the recent experiments with the use of cryoelectronic microscopy to visualize the process of membrane fusion mediated by the HA [32,33,34] revealed a structure notably different from stalk: the so-called π-shaped structure. In this configuration, a through pore is likely to exist in one of the membranes, with the HA fusion peptides aligned along the edge of the pore (Figure 1b). The membrane is strongly convex, bulging towards the other membrane, into which the HA transmembrane domains are anchored. The pore is assumed to be formed due to membrane deformation caused by interaction with the fusion proteins. In particular, the structure of the fusion protein domains was hypothesized to impose considerable elastic stress on the relatively flat membrane surface, whereas in the strongly bent edge of the pore the elastic tensions can be relieved [29]. It seems reasonable to assume that the structure of fusion proteins would determine the choice of the fusion scenario. In the present work, we investigate this correlation and try to identify the parameters of the fusion proteins that play a definitive role in the selection of one of the alternative trajectories of the membrane fusion process. We also explore the ability of the membranes to fuse in case a π-shaped structure is formed after the conformational transition of the fusion proteins.

Ostensibly, the diversity of the fusion process pathways cannot be related to the structure of the transmembrane domains of the fusion proteins anchored into the viral membrane. First of all, their structure is rather simple and conservative. As a rule, the TM domains are formed by a single α-helix, the length of the hydrophobic part of which roughly coincides with the hydrophobic thickness of the membrane (or slightly exceeds it) [2]. The viral or virus-like particle membrane containing the TM domains of fusion proteins remains almost completely undeformed in the experiments. This should largely suppress (inhibit) the responsiveness of the fusion process to any changes of structure or conformation of the TM domains. The fact that the domains are usually separated from the point of initial contact of the fusing membranes by a considerable distance makes the influence of their precise structure and physical state on the trajectory and fine regulation of the fusion process even less plausible.

It is the interaction of the peptides with the target membrane that appears to play a primary role in this context. Point mutations of the fusion peptide are known to be capable of causing the appearance of pores in the membrane or complete inhibition of fusion [35,36,37,38]. The fusion peptides of different proteins can differ in the mechanisms of their incorporation into the membrane. The HA fusion peptides are known to form boomerang-shaped α-helices, incorporating into the host cell membrane at a small depth and small angle [37,39]. It is also experimentally verified that they can be incorporated into the membrane approximately at the right angle and pierce an entire monolayer [40]. HIV fusion peptides can form either α-helices or β-sheets in the bilayer lipid membranes, depending on the membrane composition [41]. According to [42], the depth of insertion of the HIV fusion peptide into the membrane correlates positively with the effectiveness of the fusion process. In recognition of the available information about interaction of different fusion peptides with the target membrane, we consider peptides of different sizes and take into account the effects of different depths of peptide insertion into the membrane.

The fusion event usually involves several fusion proteins ensuring tight contact between the membranes by cooperatively applying mechanical force to them. A cluster of fusion proteins is termed a fusion rosette. In case of the influenza virus, the fusion rosette consists of three to eight HA proteins [20,21,22]. It can be deduced from the results reported in [28] that the appearance of the leaky structures can be caused by an improperly assembled fusion rosette, and that special proteins exist that can surround the leak location to prevent expansion of the pore and death of the cell. It also implies that the size of the protein rosette can influence the process of pore formation in the target membrane.

We consider two possible scenarios for the system behavior: membrane fusion through stalk formation, and the occurrence of a pore in one of the membranes with formation of a π-shaped structure. For each of the scenarios, we calculate the total energy of the system consisting of the two membranes. In order to calculate the deformational component of the energy, we employ an adaptation of the liquid crystal elasticity theory to lipid membranes (for detailed description of the methodology see, e.g., [43,44,45]). The membrane is treated as a continuous medium subject to elastic deformations. The sources of deformations are the fusion protein domains incorporated into the membrane. The membranes are subdivided into several parts so that deformations in each of them can be deemed small. It is assumed that, for each fixed configuration of proteins, the membrane adopts the state with the minimal energy for the given boundary conditions. The steady state configurations are found by means of minimization of the membrane elastic energy functional. The functional variation yields the Euler–Lagrange differential equations, solution of which with the appropriate boundary conditions determines the energy of each part of the membrane. The boundary conditions are determined by the topology of the membrane parts and the geometry of incorporation of the fusion proteins into the membrane. Thus, the membrane elastic energy is determined as a function of the membrane parameters and boundary conditions. Besides the energy of the membrane deformations, we take into account the energy of hydration and hydrophobic interactions between the membranes, the contribution of which becomes significant at small distances. Thus, we obtain the dependence of the total membrane energy on the geometric parameters—the distance between the membranes and the radius of pore in one of them— while the energy is minimized with respect to all other degrees of freedom. This allows the determination of the height of the energy barrier that has to be crossed by the system to form a stalk or a pore. The geometric characteristics of the fusion peptide complexes are varied in order to evaluate the sensitivity of the energy barrier height to their changes. Comparison of the resultant heights of the energy barriers for each of the scenarios allows making judgement about the relative likelihood of each of them.

## 2. Results

**Figure 2:**
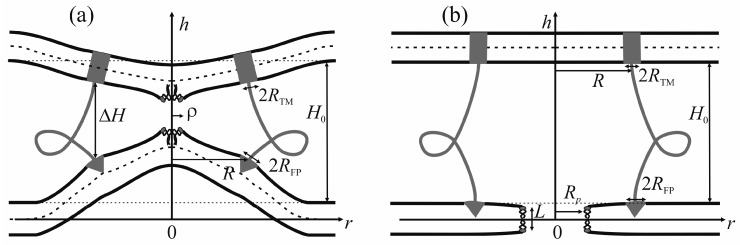
Schematic representation of the two alternative scenarios of evolution of the modeled system. (**a**) Stalk-mediated fusion: the distance ∆*H* between the fusion peptides and the transmembrane domains of the fusion proteins in the membranes is assumed as the reaction coordinate; (**b**) Formation of the dead-end state (π-shaped structure): the reaction coordinates are the pore radius *R_p_* and the length *L* of the hydrophobic part of the pore. The transmembrane domains (with the half-width of *R*_TM_) are schematically shown as gray rectangles, the fusion peptides (with the half-width of *R*_FP_) are represented by gray triangles. *H*_0_ is the equilibrium distance between the membranes, *ρ* is the radius of the hydrophobic spot formed in the area of maximal proximity of the membranes.

The viral particle and host cell membrane curvature (~50 nm) greatly exceeds the characteristic length of decay of deformations (~1 nm), and hence herein we assume the membranes to be planar away from the incorporated fusion proteins. We consider two planar parallel bilayers with incorporated fusion proteins. The membrane housing the TM domains of the fusion proteins will be termed the viral membrane, and the membrane into which the fusion peptides are incorporated will be referred to as the target membrane. The membranes are assumed to be horizontal, with the viral membrane on the top (Figure 2). The fusion protein rosette is considered as a continuous annular structure. The TM domains and fusion peptides are modeled by two coaxial rings with the radius of *R* and the half-widths of *R*_TM_ and *R*_FP_, respectively. The TM domains are modeled as annular inclusions piercing the entire depth of the bilayer of the viral membrane, whereas fusion peptides—as annular inclusions incorporated into one of the monolayers to a certain depth depending on the specifics of the peptide structure (see Figure 2). For trimeric configuration of fusion proteins, the annular structure of fusion peptides can be formed by aligning two peptides of each trimer along the fusion rosette circle, and directing the third peptide outward the circle center. Let us introduce a cylindrical coordinate system *Ohr*, with the origin *O*, the axis *Or* lying in the plane of the inter-monolayer surface of the bottom membrane, and the axis *Oh* along the rotational symmetry axis of the system. Due to cylindrical symmetry, the system is effectively unidimensional, i.e., all the values only depend on *r*.

We assume that the fusion proteins bring the membranes to close apposition at a certain distance *H*_0_, at which the attraction force imposed by the proteins is equilibrated by the repulsion forces induced by membrane hydration [46]. Further evolution of the system can only be driven by thermal fluctuations of the lipid bilayers. Two possible trajectories of the system evolution corresponding to different modes of interaction of proteins with the membrane are considered. In the first case, the membranes are brought into tight contact at the expense of conformational transitions of the fusion proteins causing juxtaposition of their transmembrane domains with the fusion peptides. It is assumed that, in the course of fusion, the distance is ∆*H* between the ring of the transmembrane domains in the viral membrane and the ring of the fusion peptides in the target membrane, while the distance between the membranes away from the fusion rosette remains equal to *H*_0_ (see Figure 2a). An energy barrier associated with hydration-induced repulsion has to be crossed in order to bring the membranes in close proximity [47]. It is assumed that, under the conditions when fusion proteins attempt to bring the membranes in juxtaposition, strong hydration repulsion results in lateral displacement of the lipid head groups from the area of contact of the membranes [6]. Thus, hydrophobic defects are generated in the contacting monolayers of the merging membranes [48]; the radius of the hydrophobic defect is designated as *ρ* on Figure 2a. Such defects can serve as monolayer fusion nucleation centers, since their formation induces local loss of order of the hydration layers and appearance of hydrophobic attraction between them [49]. The attraction between hydrophobic defects in the contact monolayers of the merging membranes leads to stalk formation (Figure 1a).

An alternative theory of the fusion process is associated with spontaneous formation of a transversal pore. The fusion peptides are assumed to partition preferentially into the vicinity of the pore (Figure 2b), because incorporation of the peptides generally leads to the target membrane deformation in relation to the unperturbed planar initial state. The membrane at the edge of the pore is also strongly deformed, and preferential partitioning of the peptides into this area alleviates the elastic stress associated with the both the deformations around the peptide and the deformations in the vicinity of the pore. This possibility is conditional to the peptide lateral mobility allowing them to choose the preferred localization. Occurrence of the hydrophobic zones capable of providing the attraction needed to juxtapose the merging membranes is believed to be hindered in the π-shaped structure configuration (Figure 2b). As opposed to the stalk configuration, formation of hydrophobic zones in the vicinity of the center of the fusion rosette is impossible in the π-shaped structure case as there is a pore in the bottom membrane. Hydrophobic regions can only occur if the fusion peptides remain localized away from the edge; in this case, formation of radially symmetrical hydrophobic belts is likely to be possible. However, the energy associated with formation of such belts would be much higher than the energy of formation of a compact circular defect in the area of tight contact between the membranes because the perimeter of the annular belt has to exceed the perimeter of the hydrophilic pore, which cannot be smaller than ~15 nm (corresponding to the pore radius of ~2 nm) for purely geometric reasons. In principle, a compact hydrophobic region can be formed at the edge of the pore, with a violation of the rotational symmetry of the system. However, in this case, the mutual approach of the membranes in the area of the hydrophobic region is likely to be hindered more than in the case of radially symmetrical stalk due to proximity of the fusion peptides to the region. We therefore contend that, in the π-shaped structure configuration, apposition of the membranes cannot end up in their fusion, and this trajectory of the process leads to a dead end state. As we demonstrated earlier, a hydrophilic pore in the membrane can be formed through an intermediate state of a hydrophobic defect [44,45,50]. In this state, lipid molecules shift radially in the plane of the membrane to form a water-filled cylinder piercing the entire membrane (Figure 2b). The side surface of the cylinder is formed by the carbohydrate chains of lipid molecules. As the hydrophobic defect expands, polar headgroups of lipids “slip” into the lumen of the cylinder, ultimately forming a hydrophilic pore [44,45,50]. It takes crossing an energy barrier associated with the formation of a cylindrical hydrophobic defect, reorientation of the lipid molecules required to form the hydrophilic pore, and possibly a redistribution of the fusion peptides towards the pore edge.

For each of the two scenarios, we calculated the dependence of the total energy *W*_T_ of the membranes upon the process coordinate. In the case of stalk, the coordinate is the change of distance ∆*H* between the fusion peptides and the transmembrane domains of the fusion proteins located in the target membrane and in the viral membrane, respectively (Figure 2a), while the radius of the hydrophobic patch *ρ* can vary freely. As we demonstrated earlier, the process of formation of a pore through the membrane is described by at least two coordinates [44,45,50,51]. In the case of the π-shaped structure, we selected the height of the hydrophobic part of the pore *L*, and the pore radius *R_p_* (Figure 2b) as the system coordinates. The energy contributors vary significantly between the two scenarios.

In the calculations, we assume the values of the elastic moduli typical for “common” lipids; based on the experimental and theoretical results these are *B* = 10 *k_B_T* and *K* = 10 *k_B_T*/nm^2^ [52,53] for leaflet splay and tilt moduli, respectively. The monolayer surface tension *σ* is assumed equal to 0.01 *k_B_T*/nm^2^. The equilibrium thickness of the monolayer *h*_0_ is assumed at 1.5 nm, which is consistent with the values observed for 1,2-dioleoyl-sn-glycero-3-phosphocholine, 1,2-dioleoyl-sn-glycero-3-phosphoethanolamine, 1-palmitoyl-2-oleoyl-sn-glycero-3-phosphocholine etc. For hydration-induced repulsion parameters, we used the following values from the work [54]: disjoining pressure *P*_0_ = 60 *k_B_T*/nm^3^, characteristic length of hydration repulsion *ξ_h_* = 0.35 nm. The characteristic length of hydrophobic attraction *ξ_f_* is considered equal to 1 nm [49]. The equilibrium distance between the fusing membranes *H*_0_ is assumed at 5 nm. This distance reflects steric interactions associated with the fusion protein ectodomains that have to be accommodated between the membranes. As the membranes are brought together, the HA protein undergoes a conformational rearrangement [18], in the course of which one of the parts of the HA ectodomain, namely HA1 subdomain, moves away thereby decreasing the effective diameter of the ectodomain just to the diameter of HA2 subdomain, which is about 5 nm [18]. Note that the energy of the π-shaped structure is independent on the intermembrane distance, while the stalk and hemifusion diaphragm energy grows up for increasing distance [55]. The half-width of the TM domain *R*_TM_ is assumed at 1 nm.

**Figure 3:**
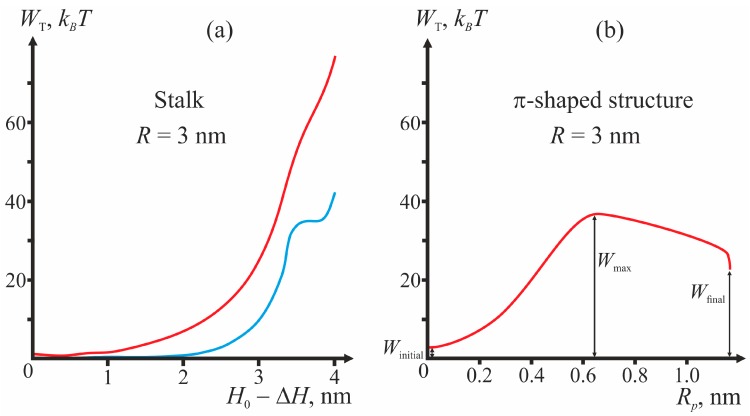
Dependence of the total energy (*W*_T_) of the membrane on the process coordinates for the scenarios with formation of stalk (**a**); and π-shaped structure (**b**). The geometric parameters are as follows: the radius of fusion rosette *R* = 3 nm, half-width of the fusion peptide annulus *R*_FP_ = 0.1 nm, shallow insertion. In the case of stalk, the red curve corresponds to the total energy *W*_T_, the blue curve—to the sum (*W_h_* + *W_f_*), i.e., the total energy less the deformation energy. For the case of π-shaped structure, only the dependence on the pore radius *R_p_* is shown; the energy is minimized with respect to the hydrophobic belt height *L* for each fixed *R_p_*.

We vary the parameters determining the geometry of fusion proteins, and for each set of parameters calculate the energy barriers corresponding to the transitions of the membrane with the incorporated peptides to the stalk and π-shaped structure configurations. Figure 3 presents the plots of dependencies of the total energy on the coordinates of the process illustrating the algorithm of calculations of the energy barrier for fixed geometry of fusion proteins. For the case of the π-shaped structure, dependence upon only one coordinate, pore radius *R_p_*, is presented. The height of the hydrophobic part of the pore *L* also varies in this process from *L* = 3 nm corresponding to the equilibrium thickness of the bilayer 2*h*_0_, to zero. The vertical drop of energy at the point *R_p_* = 1.1 nm of the plot corresponds to change of the height of the hydrophobic belt *L* at constant pore radius *R_p_*.

As illustrated by Figure 3, in the initial state of the system, the energy is not equal to zero, since the incorporation of TM domains and fusion peptides causes deformation of membranes. For stalk, the end state of the system (∆*H* = 0) can correspond to a local minimum or a maximum of total energy (see, e.g., Figure 3a). In the latter case, the calculated height of the energy barrier is only a lower-bound estimate, since further expansion of stalk towards formation of hemifusion diaphragm cannot be considered in the framework of our model and can cost additional energy. In Figure 3b, the points corresponding to the system’s initial state (*W*_initial_), final state (*W*_final_), and energy maximum (*W*_max_) are shown.

**Figure 4:**
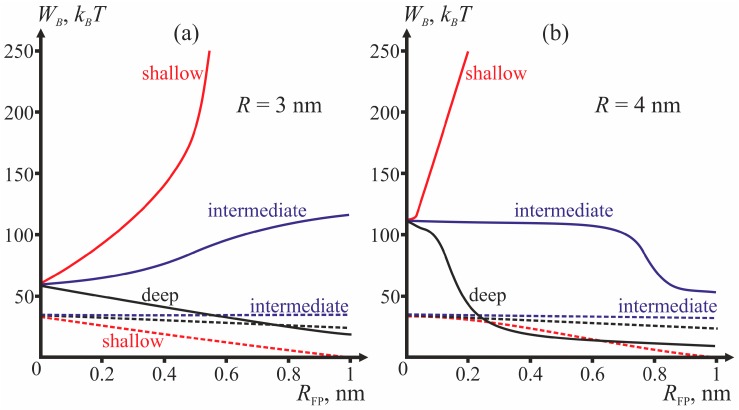
Dependencies of the height of the energy barrier (*W_B_*) for formation of the stalk (solid curves) and the π-shaped structure (dashed curves) on the half-width of the fusion peptide *R*_FP_. (**a**) fusion rosette radius *R* = 3 nm; (**b**) fusion rosette radius *R* = 4 nm. The red curves correspond to shallow insertion, the blue curves—to intermediate insertion, and the black curves—to deep insertion.

Comparing the energy barriers for system transitions into the stalk and π-shaped structure configurations, we determine for each geometry of fusion proteins the possibility of membrane fusion (stalk formation). We vary three geometric parameters: fusion rosette radius *R*, fusion peptide half-width *R*_FP_, and the depth of incorporation of the fusion peptide. The cases of *R* = 3 and *R* = 4 nm are analyzed for three different depths of incorporation of the fusion peptide (shallow, intermediate and deep insertion); the half-width of the fusion peptide is varied from 0 to 1 nm. Figure 4 presents dependencies of energy barriers on *R*_FP_ at fixed *R* for the three depths of insertion. Figure 4a corresponds to *R* = 3 nm, Figure 4b—to *R* = 4 nm.

**Figure 5:**
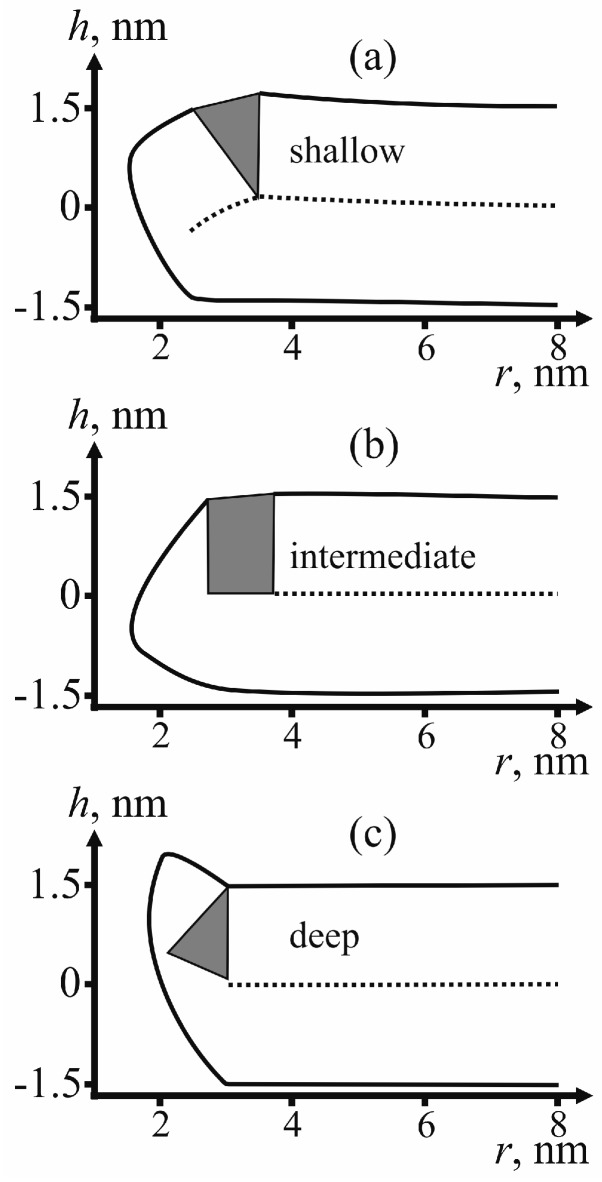
Calculated shape of the membranes for different depths of insertion of fusion peptides. (**a**) Shallow insertion; (**b**) intermediate insertion; (**c**) deep insertion. Fusion peptide is shown in gray, the dotted line is the position of the inter-monolayer surface.

For determining the state of fusion peptides in the configuration of π-shaped structure, we calculate the membrane surface shape for each type of insertion: shallow, intermediate and deep. Figure 5 provides examples of the membrane surface shape corresponding to the fusion rosette radius *R* = 3 nm and the half-width of the fusion peptide *R*_FP_ = 0.5 nm.

According to Figure 5, no displacement of peptides to the equator of the pore edge occurs for any depth of their insertion. To verify that the structure is indeed a dead end, we have to analyze possibilities for its further evolution. One option is closure of the pore with return of the system to the initial state. To achieve it, the system has to go back across the energy barrier. The energy barrier to reach the pore state is defined as the difference of the maximal and the initial energy on the trajectory (see Figure 3b):

*W_B_* = *W*_max_ − *W*_initial_ (1)

The reverse transition energy barrier is the difference of the maximal and the final energy:

*W*_RB_ = *W*_max_ − *W*_final_ (2)

Thus, the reverse barrier can be found from the direct transition barrier as follows:

*W*_RB_ = *W_B_* + *W*_initial_ − *W*_final_ (3)

**Figure 6:**
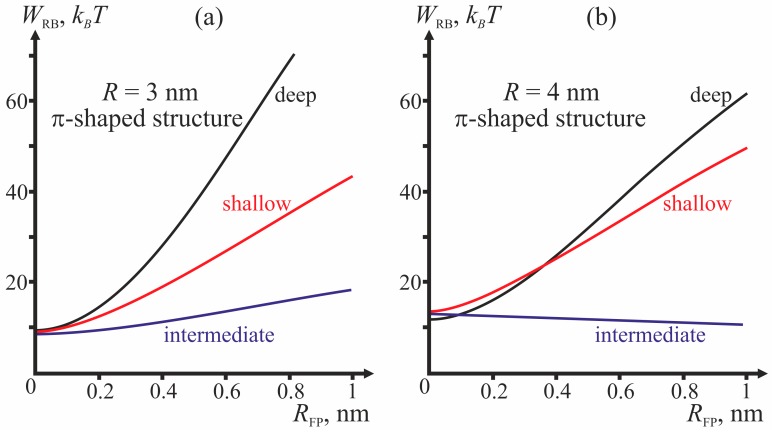
Dependence of the energy barrier for transition from the π-shaped structure to the initial unperturbed state (*W*_RB_) on the half-width of the fusion peptide *R*_FP_ for different fusion rosette radii. (**a**) *R* = 3 nm; (**b**) *R* = 4 nm. The red, blue and black curves correspond to shallow, intermediate and deep insertion, respectively.

The dependencies of the reverse barrier on the half-width of the fusion peptide for the cases of *R* = 3 and *R* = 4 nm are shown on Figure 6.

Besides the pore “healing” option, a possibility of fusion of the membrane containing the pore with the virus membrane also needs to be considered. For the membrane fusion to take place, hydrophobic defects need to form in the membranes. Due to the presence of the pore in the bottom (target) membrane, the hydrophobic region in it can only be formed along the annulus in the vicinity of the fusion peptide ring. However, the energy barrier associated with the formation and evolution of an annular hydrophobic region is too high to be crossed at the expense of thermal fluctuations. We evaluate it based on the energy barrier calculated for the case of stalk-mediated fusion. As shown in Figure 3a, the energy not associated with membrane deformations peaks at 40 *k_B_T* (blue curve in Figure 3a). The hydrophobic patch radius at that point is ~1 nm. This means that, in order to create a hydrophobic defect with the area of 1 nm^2^, the system has to cross the energy barrier exceeding 12 *k_B_T*. According to Figure 5, the inner radius of the hydrophilic pore is ~1 nm. We consider that for fusion, the width of the hydrophobic belt must also be no smaller than ~1 nm. Its area then exceeds 18 nm^2^. It means that the energy barrier associated with formation of such a belt is larger than 100 *k_B_T* and cannot be crossed at the expense of thermal fluctuations within the characteristic time of fusion (~1 min) [6].

## 3. Discussion

It is presently believed that, in most cases, membrane fusion occurs through no-leak mechanisms, implying formation of a stalk [9]. Pore formation in the course of fusion was believed to be a pathological process and was not systematically investigated. However, in a number of recent experimental works [27,34,35], the existence of the fusion structure with pores was demonstrated. Molecular dynamics simulations confirm that fusion proteins can facilitate formation of pores in the membrane [29]. Fusion proteins are also known to play a crucial role in crossing of the energy barrier associated with stalk formation [9,56]. It implies that, in certain cases, fusion proteins facilitate membrane fusion, whereas in other cases the formation of the structures does not lead to such fusion, and the latter option is not exceptionally rare. We analyzed such structures and the characteristics of the fusion proteins essential for evolution of the fusing membrane.

The most important parameter for the choice between the stalk and the π-shaped structure scenario is the structure of fusion peptides and geometry of their incorporation into the host cell membrane. In our model, this structure is described by two parameters: half-width *R*_FP_ and depth of insertion, i.e., the value of the boundary director. According to Figure 4, the behavior of the system is determined by the depth of incorporation of the peptide: decreased depth of incorporation hinders fusion due to increase of the energy barrier to stalk formation. Pore formation proves the least favorable in the case of intermediate depth of insertion and the most favorable in case of shallow insertion. The larger the half-width of the fusion peptide, the more favorable is pore formation. For the biologically relevant sizes of the fusion peptide (*R*_FP_ ≥ 0.5 nm), shallow incorporation of the fusion peptide facilitates formation of the π-shaped structure, and deep insertion facilitates membrane fusion. In the case of intermediate incorporation, the fusion rosette radius *R* plays a definitive role: the larger the fusion rosette, the more favorable stalk formation becomes.

As can be seen from the calculated shape of the membrane (see Figure 5), the peptides do not relocate towards the equator of the pore. The pore radius in these conditions is about 1 nm. This result is consistent with the previously obtained data on dependence of lipid pore energy on its radius [44,45,50]. According to these calculations, the energy of lipid pore has a minimum corresponding to the radius of ~1 nm; further increase of the radius results in abrupt increase of the deformation energy of the membrane. Due to the presence of hydrophilic pore in a membrane, fusion of the membranes is hindered by the need to form a large hydrophobic area with energy of about ~100 *k_B_T*. The reverse barrier corresponding to pore closure depends on the depth of incorporation of the fusion peptide (see Figure 6). In the case of intermediate depth, it is almost insensitive to the changes of half-width *R*_FP_ and amounts to about 10–15 *k_B_T*, i.e., it can be relatively rapidly crossed at the expense of thermal fluctuations. In other cases, for biologically adequate sizes of the fusion peptide (*R*_FP_ ≥ 0.5 nm) the energy barrier would exceed 50 *k_B_T*, i.e., too high to be crossed at the expense of thermal fluctuations in reasonable time. Therefore, π-shaped structure turns out to be a dead end final state in the case of shallow and deep insertion: both fusion and return into the initial state are hindered. In the case of intermediate depth of incorporation, a return to the initial state proves possible. Thus, in the case of intermediate depth a scenario with periodic opening and closure of a pore in the target membrane preceding stalk-mediated fusion is possible. These reversible changes, as well as a significant height of the stalk formation energy barrier (~50 *k_B_T*) must substantially slow down fusion of membranes in case of the intermediate depth of insertion of fusion peptides.

Our results offer explanations for a number of experimental findings. Thus, it was demonstrated in the work [42] that there is a correlation between the depth of incorporation of fusion peptide of HIV and the effectiveness of fusion mediated by the peptide: the deeper the insertion, the more effective the membrane fusion. Similar correlations were observed in a number of works dedicated to analysis of mutants of influenza virus.

It is broadly assumed that the boomerang-shaped HA fusion peptide incorporates deeply into the contact monolayer of the target membrane [38]. It was demonstrated that a mutation transforming the fusion peptide into α-helix lying on the surface of the membrane completely inhibits fusion [37]. A mutation causing an increase of the angle between parts of the boomerang-shaped peptide, and hence partial decrease of the depth of its insertion, results in a decrease of the fusion effectiveness [35], with a leak observed immediately before the fusion. Our results explain both the decreased fusion effectiveness and the observed leak. Insignificant decrease of the depth of incorporation in our terms means that the fusion peptide is now incorporated to an intermediate depth. According to our results (see Figure 4), intermediate insertion can facilitate opening of the pore with subsequent rapid closure. It means that in the case of intermediate insertion, occurrence of leak is possible before the fusion. In case of shallow insertion, no fusion occurs.

The obtained results are indicative of a generic nature of the correlation between the depth of insertion of the fusion peptide and effectiveness of fusion, and allow concluding that the deeper fusion peptide inserts into the membrane of the target cell membrane, the more effectively fusion occurs. We failed to find any credible correlation between the hydrophobic to hydrophilic residue ratio and the depth of insertion. One of the reasons for that is very low variability of this ratio associated with the known mutations of fusion peptides. More generally, the hydrophobicity/hydrophilicity of individual amino acids, often quantified through “hydrophobic (or lipophilic) potential” or other similar measures, is a predominantly entropic phenomenon determined by the ability of the molecule to form hydrogen bonds and shift electronic density within its moieties to minimize the overall electrostatic energy. Intramolecular H-bonding reduces the apparent hydrophilicity of the molecule by leaving fewer H-bond donor/acceptor groups for interactions with water [57]. Likewise, an amino acid residue as a part of a peptide or protein molecule can form hydrogen bonds or enter hydrophobic interactions with its neighbors or other proximal residues, thereby reducing its effective hydrophilicity or hydrophobicity score. Thus, a residue of an amino acid behaving as hydrophilic in isolation can be a part of a highly hydrophobic domain by virtue of sequestration of the residue’s polar moieties via intramolecular interactions. As a side note, the occurrence of polar residues within transmembrane domains often drives their oligomerization, the residues serving as oligomerization interfaces, in which case the interactions are intermolecular. Thus, membrane-embedded domains, including fusion peptides, can and do accommodate multiple “hydrophilic” residues. The energy cost of bringing a charged residue into the hydrophobic core is usually prohibitively high, but even such examples, though rare, are not totally unheard of. Accordingly, as stated in the review [23], it is the fusion peptide primary and secondary structure rather than a sum of properties of individual amino acid residues that determines the depth of insertion of the peptide. Their variance thus provides an instrument for fine regulation of the fusion effectiveness.

For the purpose of this analysis, we assumed homogeneous distribution of lipids. Cellular membranes are known to consist of multiple lipid species with highly heterogeneous lateral distribution, including formation of microdomains of liquid ordered (L_o_) phase (“rafts”) with the local lipid composition different from that of the bulk lipid [58]. The liquid ordered domains are also known to be thicker than the surrounding membrane [59], which results in line tension of the domain boundary [43]. According to recent experimental studies, the raft boundary structure plays an important role in the process of viral fusion [60]. In particular, the HIV fusion peptide tends to be preferentially inserted along the raft boundaries, and phase separation with formation of such boundaries substantially enhances fusion efficiency. By contrast, no enhancement of fusion efficiency was observed in the membranes consisting exclusively of the L_o_ phase without any phase separation boundaries. These trends, however, do not apply to the influenza virus. This difference in the behavior of the HIV and influenza virus fusion peptides can be attributable to different depths of insertion of the corresponding fusion peptides into the host cell membranes. A correlation can be found between the difference in the insertion depths and the difference in the spontaneous curvatures induced by the insertion: deeper penetration (as in case of the HIV) is similar to induction of negative spontaneous curvature, whereas more shallow incorporation (e.g., influenza virus) induces nearly zero spontaneous curvature. According to our prior results [59], membrane components with non-zero spontaneous curvature (regardless of the sign) preferentially distribute into the phase separation boundaries, so that such boundaries can serve as local concentrators of the fusion peptides with deep insertion and define their orientation in the membrane. Besides that, regardless of the preferences of the fusion peptides, the phase separation boundary has excess energy that can be characterized by the line tension of the boundary and is proportional to its total length. One of the viable options for minimizing the boundary energy is to bend the domain surface to form a hemispherical bulge protruding from the membrane plane. If it happens in the area between the viral membrane and the host cell membrane, minimal distance between the membranes would decrease, facilitating the fusion process through stalk intermediate. Thus, formation of a domain with a high line tension of the boundary should further facilitate fusion. We suppose that incorporation into the target membrane of the fusion peptides with preference towards the phase separation boundary can serve to nucleate domain formation in the target membrane. In other words, under appropriate conditions the fusion peptides can induce formation of phase separation boundaries rather than preferentially partitioning into the pre-existing boundaries. A detailed theoretical model of the process of fusion of phase-separating membranes is an intended topic of our further investigations.

## 4. Materials and Methods

### 4.1. Stalk Energy

In the case of stalk, the free energy is represented by a sum of the membrane elastic deformation energy *W_e_*, the energy of hydration repulsion between the membranes *W_h_*, and the energy of interaction between hydrophobic defects in different membranes *W_f_*:

*W*_T_ = *W_e_* + *W_h_* + *W_f_*. (4)

The deformation is treated according the Hamm–Kozlov model [53], which has proven itself in the description of membrane processes [43,59,61,62]. A field of unit director vectors **n** characterizing the average orientation of lipid molecules is introduced for description of the membrane monolayer deformations. The vector field is defined on a certain surface inside the monolayer parallel to its external boundary, known as a dividing surface. The shape of the surface is defined by a field of unit vectors **N** normal to it; the normal vectors are considered to be directed towards the inter-monolayer surface of the membrane. We consider only the following two main deformation modes: tilt and splay. We assign all deformations and elastic moduli to a specific dividing surface, so-called neutral surface, defined as the surface where energy contributions from splay and lateral stretch/compression deformations are independent of each other. According to the experimental results of [63], the neutral surface is situated in the area of junction between the polar head groups and acyl chains, at the depth of ~0.5 nm from the outer monolayer boundary. The splay deformation is quantitatively described by divergence of the director over the dividing surface, whereas tilt deformations are described by the tilt vector **t** = **n**/(**nN**) − **N** ≈ **n** − **N**. If the deformations are assumed small and zero energy is assigned to the undeformed planar bilayer, the energy of deformed monolayer can be expressed as [53]

(5) $$W=\int \left(\frac{B}{2}{\left(\mathrm{div}n\right)}^{2}+\frac{K}{2}t^{2}+\sigma \right)dS-\sigma A_{0}.$$

*B* and *K* in this expression are splay and tilt moduli, respectively; *σ* is monolayer lateral tension; *dS* is the elementary area of the neutral surface; *A*_0_ is the neutral surface area in the initial undeformed state. Smallness of deformations means that the projection of the director on the axis *Or* is much smaller than unity. All vectors are replaced with their projections on this axis. In order to define the state of two monolayers in the membrane, five functions need to be introduced: projections of the directors of the upper and the lower monolayer on the *Or* axis, *a*(*r*) and *b*(*r*), respectively; distance from the *Or* plane to the neutral surfaces of the upper and lower monolayers, *h_a_*(*r*) and *h_b_*(*r*), respectively; distance from the *Or* plane to the inter-monolayer surface, *m*(*r*). Besides that, we assume the hydrophobic zone of the monolayer to be locally volumetrically incompressible, i.e., that the volume of any element of the monolayer does not change upon deformation. This assumption is justified by a large value of the volumetric compressibility module. The local incompressibility condition is written as follows [53]:

(6) $$\Delta h=h_{0}-\frac{h_{0}{}^{2}}{2}{\left(\mathrm{div}n\right)}^{2},$$

where Δ*h* is the width of the monolayer in the given point of the *Or* plane; *h*_0_ is the thickness of undeformed monolayer. Equation (6) in combination with the definitions of the tilt vector (**t** = **n** − **N**), monolayer thickness (∆*h_a_* = *h_a_*(*r*) − *m*(*r*), ∆*h_b_* = *m*(*r*) − *h_b_*(*r*)), and normal to the neutral surface of the monolayer (**N_a_** = **grad**(*h_a_*(*r*)), **N_b_** = −**grad**(*h_b_*(*r*))), relates the tilt vectors *t_a_*(*r*) and *t_b_*(*r*) in the upper and lower monolayer with the directors *a*(*r*) and *b*(*r*) in these leaflets and the location of the inter-monolayer surface *m*(*r*). Thus, by imposing the conditions of local volumetric incompressibility to the two leaflets of the membrane, the number of independent functions characterizing the state of the membrane can be reduced from five (*a*(*r*), *b*(*r*), *h_a_*(*r*), *h_b_*(*r*), *m*(*r*)) to three (*a*(*r*), *b*(*r*), *m*(*r*)). We express the elastic energy functional Equation (5) through these three functions, and search its extrema by varying the functional with respect to the independent functions *a*(*r*), *b*(*r*), *m*(*r*) and thus obtaining three Euler-Lagrange differential equations. The solutions of these equations are then substituted into the elastic energy functional Equation (5). The expressions for *a*(*r*), *b*(*r*), and *m*(*r*) obtained by solving the Euler-Lagrange differential equations contain indefinite coefficients, which are determined by minimizing the energy taking into account the boundary conditions, which are defined by the geometry of the fusion peptides, TM domains and hydrophobic regions in the contact monolayers. More detailed descriptions of the methods used for elastic energy calculations are provided in the works [43,61,64,65].

**Figure 7:**
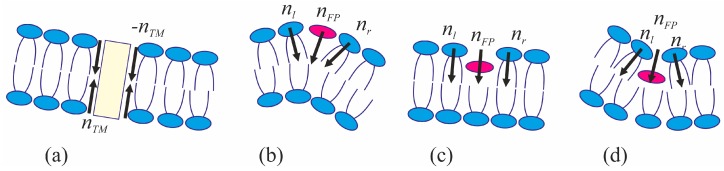
Schematic representation of a fusion peptide in a bilayer. (**a**) transmembrane domain; (**b**) case of shallow incorporation of the fusion peptide; (**c**) intermediate incorporation; (**d**) deep incorporation. The black arrows designate the directions of the boundary directors. Transmembrane domain is represented by light-yellow rectangle, fusion peptides—by red ellipses.

The tilt of the TM domain with respect to the membrane normal causes an equal tilt of the lipids contacting it and deformation of the viral membrane. Accordingly, the projection of the director of the boundary lipids on the *Or* axis *n_TM_* becomes non-zero. (Figure 7a). Besides that, the tilt of the TM domains in the fusion rosette causes relative shift of the neutral surfaces of the membrane leaflets on the outer (*r* = *R* − *R*_TM_) and inner (*r* = *R* + *R*_TM_) boundary of the ring (Figure 2a). Thus, the following boundary conditions apply:

*a*(*R* ± *R*_TM_) = −*n_TM_*, *b*(*R* ± *R*_TM_) = *n_TM_*, *h_a_*_,*b*_(*R* + *R*_TM_) − *h_a_*_,*b*_(*R* − *R*_TM_) = −2*n_TM_R*_TM_. (7)

Fusion peptide incorporation into the membrane results in lateral displacement of parts of the contacting lipid molecules. Generally, the boundary lipids tilt by a certain angle with respect to the normal of the neutral surface of undeformed membrane. Let us designate the projection of the director on the inner boundary of the fusion peptide ring (*r* = *R* − *R*_FP_) as *n_l_*, and its value on the outer boundary (*r* = *R* + *R*_FP_) as *n_r_* (Figure 7b–d). Besides, the fusion peptide can rotate in the membrane as a whole; we denote the projection of the director describing this rotation as *n_FP_*. Obviously, *n_FP_* = (*n_l_* + *n_r_*)/2, i.e., *n_FP_* is a mean of the directors on the inner and outer boundaries of the fusion peptide ring. Using geometric interpretation of the director, the difference between the directors on the inner and outer boundaries of the fusion peptide ring (director “jump”) can be expressed through the width of the ring 2*R*_FP_ and the monolayer thickness *h*_0_ as follows:

(8) $$\begin{array}{l} a\left(R+R_{\mathrm{FP}}\right)-a\left(R-R_{\mathrm{FP}}\right)=-\frac{2R_{\mathrm{FP}}}{\sqrt{h_{0}{}^{2}+R_{\mathrm{FP}}{}^{2}}},\\ a\left(R+R_{\mathrm{FP}}\right)-a\left(R-R_{\mathrm{FP}}\right)=0,\\ a\left(R+R_{\mathrm{FP}}\right)-a\left(R-R_{\mathrm{FP}}\right)=\frac{2R_{\mathrm{FP}}}{\sqrt{h_{0}{}^{2}+R_{\mathrm{FP}}{}^{2}}} \end{array}$$

for the cases of shallow, intermediate and deep insertion of the fusion peptides, respectively (Figure 7b–d). In the cases of shallow and intermediate incorporation of the fusion peptides into the contact monolayer, a discontinuity of its neutral surface occurs. Rotation of the peptide as a whole results in relative displacement of the neutral surfaces in the inner and outer boundaries of the peptide ring:

*h_a_*(*R* + *R*_FP_) − *h_a_*(*R* − *R*_FP_) = 2*R*_FP_*n_FP_* (9)

In case of deep incorporation, continuity of the neutral surface is supposed to be maintained, and thus

*h_a_*(*R* + *R*_FP_) − *h_a_*(*R* − *R*_FP_) = 0 (10)

In the area of tight contact between the membranes, lateral fluctuation-induced displacements of polar heads of lipids of both membranes can result in formation of a circular hydrophobic region with the radius of *ρ*. The displacement is associated with membrane deformations and causes reduction of the hydration-induced repulsion energy. The lateral displacement of polar head groups alters the projections of director on the *Or* axis at the boundary of the hydrophobic region:

(11) $$a\left(\rho \right)=-\frac{\rho }{h_{0}}.$$

This condition is compatible with two limiting cases, for which the values of the boundary director are known. Indeed, symmetry considerations dictate that in case of a zero-radius hydrophobic defect (*ρ* = 0) the director at the boundary has to be zero. It is equally clear that when the radius of the defect is equal to the monolayer thickness, *r* = *h*_0_, lipid molecules are horizontal, and the director is equal to −1. Besides introducing the boundary director, we fix the distance ∆*H* between the neutral surfaces of the contact monolayers of the two membranes at *r* = *R*, and distance *H*_0_ between the neutral surfaces of the contact monolayers of the two membranes at *r* → ∞.

The energy of interaction of two planar hydrophobic regions of the contact leaflets of the viral membrane and target membrane separated by a water layer is calculated according to Israelachvili theory [49]:

(12) $$W_{f}=\sigma_{0}\pi \rho^{2}\left(1-\exp\left(-\frac{l}{\xi_{f}}\right)\right),$$

where *ξ_f_* is the characteristic length of hydrophobic interactions in water [49], *l* is the distance between the hydrophobic regions, and *σ*_0_ is the surface tension of the macroscopic boundary separating water and acyl chains of lipids. Hydration repulsion energy is calculated as described in [46,47]:

(13) $$W_{h}=P_{0}\xi_{h}\int \exp\left[-\frac{z\left(r\right)}{\xi_{h}}\right]dS,$$

where *z*(*r*) is the distance between the membranes at the given value of the radial coordinate *r, P*_0_ is the disjoining pressure characterizing the amplitude of hydration repulsion, *ξ_h_* the characteristic length of hydration interactions; the integration is performed over the hydrophilic surface of the contact monolayers. For evaluation of the integral in Equation (13), we apply Derjaguin–Landau–Verwey–Overbeek theory, according to which integration in the equation can be limited to the region in which the distance between the membrane changes by the value of *ξ_h_*, with a replacement of the deformed hydrophilic surfaces of the contact leaflets by horizontal planes. If there are no hydrophobic regions in the membranes, integration in Equation (13) starts from *r* = 0. When the membranes do contain hydrophobic regions, integration starts at *r* = *r* + *L_h_*, to make an allowance for the effect of blurring of the boundary between the hydrophobic patch and the bulk membrane. This blurring is caused by several factors—fluctuations of polar head groups of lipids (the head group size ~0.8 nm), finite persistence length of the order parameters of hydrophobic and hydrophilic interaction (~0.35 nm and 1 nm, respectively). We selected a medium value of *L_h_* ~ 0.8 nm.

### 4.2. Energy of the π-Shaped Structure

We consider formation of the π-shaped structure in the target membrane immediately after incorporation of fusion peptides when they are sufficiently far from the virus membrane, therefore we neglect the energy of the latter, as well as the hydration-induced repulsion, which starts to manifest itself at the distances between the membranes of the order of 1 nm. The energy of the π-shaped structure is represented by the sum of the membrane elastic deformation energy *W_e_*, and the energy *W_f_* of the water-filled hydrophobic cylinder piercing the target membrane (Figure 2b):

*W*_T_ = *W_e_* + *W_f_*. (14)

Hydration-induced repulsion between the membranes is disregarded in this case, since the distance between the membranes is much larger than the characteristic length *ξ_h_*. The deformation energy of the membrane with a pore is calculated based on the elasticity theory approaches, similarly to calculations of the membrane deformation energy in the stalk configuration. More details about the formalism applied are available in [44,45,50].

To remain in the framework of the linear theory, we divide the pore edge in two parts—a “horizontal bilayer” part where directors and normals weakly deviate from the *Oh* axis direction and a “vertical monolayer” part, where their deviation from the *Or* direction is relatively small. The parts are conjugated along a pair of circumferences (*R_a_*_0_, *Z_a_*_0_) and (*R_b_*_0_, *Z_b_*_0_), corresponding to two monolayers. The energy of the “horizontal bilayer” region of both viral and target membrane is calculated exactly in the same way as the energy of deformations in the case of stalk, described above.

The shape of the neutral surface of the vertical monolayer region is characterized by the distance from the rotational symmetry axis to the neutral surface, *R*(*h*), and by the projection of the unit normal to the surface onto the *Oh* axis, *N_z_*(*h*). We use designation *v*(*h*) for director projection onto the *Oh* axis. The distance from the *Oh* axis to the surface of lipid tail ends, *M*(*h*), characterizes the shape of this surface. The solutions obtained in the horizontal and vertical regions are conjugated along the circumferences (*R_a_*_0_, *Z_a_*_0_) and (*R_b_*_0_, *Z_b_*_0_) delineating them based on the considerations of continuity of neutral surfaces and director. The boundary conditions read:

*h_a_*(*R_a_*_0_) = *Z_a_*_0_, *R*(*Z_a_*_0_) = *R_a_*_0_, *a^2^*(*R_a_*_0_) − *v_a_^2^*(*Z_a_*_0_) = 1, (15)

*h_b_*(*R_b_*_0_) = *Z_b_*_0_, *R*(*Z_b_*_0_) = *R_b_*_0_, *b^2^*(*R_b_*_0_) − *v_b_^2^*(*Z_b_*_0_) = 1, (16)

where *a*, *b* are projections of the director onto *Or* axis; *v_a_*, *v_b_* are projection of the director onto *Oh* axis.

It is assumed that hydrophilic pore is formed in the originally intact bilayer through an intermediate state referred to as a hydrophobic defect. We postulate it to consist of a horizontal bilayer region, vertical monolayer region, and cylindrical hydrophobic belt of the height of *L* and radius *R_p_*, coaxial with *Oh*. The energy of water-filled hydrophobic cylinder is calculated in [51,66] based on Marcelja theory [67]. In our notation, the energy of the cylinder reads:

(17) $$W_{h}=\left(2\pi R_{p}L\right)\sigma_{0}\frac{I_{1}\left(\frac{R_{p}}{\xi_{f}}\right)}{I_{0}\left(\frac{R_{p}}{\xi_{f}}\right)}$$

where (2*πR_p_L*) is the cylinder side surface area; *I*_0_, *I*_1_ are Bessel functions of order zero and one, respectively. For the vertical region, the boundary conditions at the edge of the hydrophobic belt are stated as follows:

(18) $$\begin{array}{l} R_{a,b}\left(\pm L/2\right)=R_{p},\\ v_{a,b}\left(L/2\right)=\mp \frac{L/2}{\sqrt{{\left(L/2\right)}^{2}+{\left(h_{0}-L/2\right)}^{2}}}. \end{array}$$

The latter condition is a direct consequence of local volumetric incompressibility, i.e., constant density of lipid hydrocarbon chains, applied to the hydrophobic belt. So, the pore is parameterized by two independent variables, which are the pore radius in the equatorial plane *R_p_*, and height of the hydrophobic belt *L*.

### 4.3. Algorithm of Evaluation of the Process Trajectory Likelihood

In the calculations, we maintained fixed geometric parameters characteristic of the fusion proteins—*R*, *R*_TM_, *R*_FP_—and minimized the energy *W*_T_ with respect to all free parameters, which include the indefinite coefficients occurring in the solutions of Euler–Lagrange differential equations and geometric parameters that are not fixed, such as the radius of the hydrophobic region *ρ* or tilt of protein domains in the membrane, *n_TM_* and *n_FP_*. Thus, we obtained the dependence of the total energy *W*_T_ only on the process coordinates—∆*H* in the case of stalk, and *L* and *R_p_* in the case of π-shaped structure. Thereafter, for each of the two scenarios we determined the energy barrier, i.e., the maximal energy needed for the system to transition from the initial state (common for both scenarios) to the final state. For stalk-mediated fusion, the end state is achieved when the distal monolayers of the merging membranes form a bilayer, and ∆*H* = 0 (Figure 1a); for the case of the π-shaped structure, the end state occurs when hydrophilic pore forms in the target membrane, and *L* = 0 (Figure 1b). After that we compare the energy barriers obtained at fixed geometric parameters. We assume that the system selects a scenario in accordance with Boltzmann weight factor of the trajectory, i.e., the process with a lower energy barrier is more likely. This method allowed us to determine how the fusion protein structural properties could be modified in order to increase or decrease the membrane fusion probability.

## Abbreviations

HA	hemagglutinin
HIV	human immunodeficiency virus
TM	transmembrane (domain)
